# Missed opportunities for impact in patient and carer involvement: a mixed methods case study of research priority setting

**DOI:** 10.1186/s40900-015-0007-6

**Published:** 2015-08-04

**Authors:** R. Snow, J C Crocker, S. Crowe

**Affiliations:** 1 Health Experiences Institute, Nuffield Department of Primary Care Health Sciences, University of Oxford, Oxford, UK; 2 NIHR Oxford Biomedical Research Centre, Oxford, UK; 3 Crowe Associates Ltd., Oxford, UK

**Keywords:** Priority setting, Research priorities, Patient involvement, Patient and carer involvement, PPI, Service-user involvement, Impact, Research agenda

## Abstract

**Plain English summary:**

Healthcare workers want to listen more to patients and their carers in all sorts of areas of healthcare. This can include choosing topics for medical research. We looked at how patients and carers have helped to choose topics for research about type I diabetes. We aimed to find out if, and why, researchers often rejected their choices.

We looked at a project which brought together patients, carers and healthcare workers to choose topics for research about type 1 diabetes. The group first asked patients, carers and healthcare workers to suggest ideas for research questions.

But the group had to follow rules about what counted as a good research question. Some people’s ideas did not count as good research questions, and they were rejected at the start. We looked at who were most likely to have their ideas rejected at the start. We found that patients and carers were most likely to have a suggestion rejected. Then we looked at the rejected questions in detail. They were mostly about curing diabetes, preventing diabetes and understanding how diabetes works. There were also some questions about access to medicines and the quality of care.

Researchers should ask patients and carers for help deciding what counts as a good research question from the start of projects like these. We should also think about what might be getting in the way of patients and carers making more of a difference in research.

**Abstract:**

**Background**

Patients and carers are increasingly involved in deciding on topics for medical research. However, so far, it has been difficult to gain an accurate picture of the impact of such involvement because of poor reporting and evaluation in published studies to date. This study aimed to explore how a partnership of patients, carers, healthcare professionals and organisations identified questions for future research and why patients and carers had a limited impact on this process.

**Methods**

In the first stage of the partnership process, relevant service users and providers (including patients, carers, healthcare professionals and voluntary organisations) were invited to submit suggested research questions about the treatment of type 1 diabetes, via a national online and paper survey. The partnership followed formal protocols that defined a researchable question. This meant that many respondents’ suggested research questions were rejected at the start of the process. We analysed survey submissions to find out which groups of respondents were most likely to have their suggestions rejected and what these suggestions were about.

**Results**

Five hundred eighty-three respondents submitted 1143 suggested research questions, of which 249 (21.8 %) were rejected at the first stage. Respondents with lived experience of this long-term condition (patients and carers) were more likely than those without lived experience to submit a research question that would be rejected (35.6 vs. 16.5 %; *p* < 0.0005). Among the rejected questions submitted by patients and carers, there were several key themes: questions about cure, cause and prevention, understanding the disease, healthcare policy and economics.

**Conclusions**

In this case study, early decisions about what constituted a researchable question restricted patients’ and carers’ contributions to priority setting. When discussions about a project’s remit take place before service users are involved, researchers risk distorting the potential impact of involvement. Impact assessments should consider not only the differences patients and carers make to research but also the differences they *could* have made in the absence of systemic barriers. We recommend that initiatives aimed at involving patients and carers in identifying research questions involve them as early as possible, including in decisions about how and why suggested research questions are selected or rejected.

**Electronic supplementary material:**

The online version of this article (doi:10.1186/s40900-015-0007-6) contains supplementary material, which is available to authorized users.

## Background

A major cause of medical research ‘waste’ is lack of attention to the real-world needs of those who would benefit from research. This can be seen in the apparent mismatch between research agendas and the expressed needs of patients and carers [1, 2]. There have been calls for this gap to be closed by inviting patients and carers to help shape research priorities [1]. Similarly, it is argued that if healthcare services are to deliver patient-centred care, then the evidence base provided by research needs to be more reflective of service users’ needs and concerns. Involvement of service users in identifying and prioritising medical research questions and topics should help to ensure that research being conducted is relevant to them. Such involvement can broaden the scope of a proposed research agenda [2–4] and help to ensure that it is grounded in the day-to-day reality of service users’ experiences [5].

In recent years, there has been a growth of academic literature (largely written by researchers) reporting attempts to involve service users in identifying and prioritising research questions [5, 4]. There is no agreed best practice for involving service users in identifying priorities [6]; thus, a diverse range of processes is reported, including focus groups, postal surveys, online questionnaires, social media forums, citizens’ juries, workshops and informal communication [2, 3, 6–13]. The increasing use of formal methods, however, provides growing opportunities for evaluations to investigate participants’ influence [14] and the barriers to and facilitators of such influence. Such evaluation would help meet the growing demand for the impact of public involvement in research to be measured in order to better understand what works for whom, in what circumstances and why [15]. Unfortunately, poor and inconsistent reporting of patient involvement and a lack of critical evaluation have made it difficult to gain an accurate picture of the impact of patient involvement in research agenda setting, as in other stages of health- and social care research [5]. In addition, there is a risk that if we only measure impact that has occurred in a process designed by professionals, we may conclude that the service users have little to contribute, when in fact their influence may be limited by the process itself. In other words, if we measure impact, are we just measuring the extent to which service users are allowed to have an impact?

In this study, we sought to evaluate the unintentional barriers to impact of service users, in this case patients and carers, on the identification of research questions during the first stage of a James Lind Alliance Priority Setting Partnership for type 1 diabetes. The James Lind Alliance (JLA), a non-profit-making UK initiative established in 2004, facilitates Priority Setting Partnerships to bring together patients, carers and healthcare professionals on an equal footing, identify unanswered questions about treatment (‘uncertainties’) which are important to both groups, work with both groups jointly to prioritise the uncertainties, and ultimately produce a ‘top 10’ list of jointly agreed uncertainties as research questions to be presented to funders [16]. The Partnerships are organised around patient conditions, such that the involved patients and carers have ‘lived experience’ of the condition under study. An increasing number of these Partnerships are being run across the UK [17], providing a means by which patients and carers can potentially influence the agenda to better address their needs.

### Aims

We used the JLA Priority Setting Partnership for type 1 diabetes as a case study to (i) explore differences between patients, carers and healthcare professionals with regard to the likelihood of their identified research questions being outright rejected (i.e. not put forward to the prioritisation stage) and (ii) explain these differences by analysing the content of questions which were rejected. We aimed to co-produce this work by bringing together our complementary expertise and experience in lived type 1 diabetes, service-user involvement practice and qualitative and quantitative research methods. We hope that our findings will increase understanding of the barriers to service users’ impact on the research agenda.

This study was fully approved by the Medical Sciences Interdivisional Research Ethics Committee, University of Oxford.

## Methods

The methods of data collection and subsequent process for this Priority Setting Partnership have been described more fully in a separate paper, which did not analyse those processes [18]. The relevant methods from this original project are summarised below.

### The Priority Setting Partnership

The Partnership involved the following organisations: Juvenile Diabetes Research Foundation, Insulin Dependent Diabetes Trust, Diabetes Research Network, Diabetes UK, Scottish Diabetes Research Network, UK Database of Uncertainties in the Effects of Treatments and the James Lind Alliance and NHS Evidence—diabetes. There were also individual members with perspectives in paediatrics and primary care, as well as users of type 1 diabetes services.

A Steering Group of nine representatives from these groups plus an independent information specialist oversaw the whole Partnership process. They met three times face to face and participated in five teleconferences throughout 2010 and 2011. The Insulin Dependent Diabetes Trust provided core funding for the project, with other partners contributing time and expertise in kind, including the production of survey materials and distribution of update reports.

### Identifying potential research questions

The first step in the Partnership process was to identify potential research questions of interest to service users and providers. A survey was developed (online and paper-based) which asked, ‘What question(s) about the treatments for type 1 diabetes would you like to see answered by research? (You can submit as many or as few as you like.)’ Questions were submitted anonymously as free text. Respondents were also asked to state their relationship to type 1 diabetes, for example, whether they had the condition, cared for someone with type 1 diabetes and/or worked as a diabetes specialist. Respondents were allowed to tick more than one box. Members of the general public, that is, those with no connection to type 1 diabetes, were not included in the exercise. Consent to publish the questions provided by the survey was requested of all respondents. Only questions from those who provided consent have been quoted in this study.

The survey was piloted with members of the steering group and their contacts, leading to several revisions before the final version was launched in 2010 (see Additional file 1). The online survey ran from 10th March to 23rd May 2010 and was freely accessible online via a SurveyMonkey web link. All member organisations of the Partnership promoted the survey and web link through widespread distribution of information sheets, posters, postcards and flyers (see Additional files 2 and 3), targeting adults and young people with type 1 diabetes, parents of children with type 1 diabetes and healthcare professionals with an interest in type 1 diabetes. The survey was also highlighted to clinicians at the Diabetes UK annual professional conference in March 2010. Respondents were recruited in this way by snowballing.

### Processing suggested research questions

Due in part to the nature of its funding, the Partnership had a strict protocol in place to ensure that the exercise would establish interventional research priorities only, i.e. those research questions that would inform treatment interventions. As a result, on completion of the survey phase, an information specialist reviewed all the submitted entries and discarded entries which were not concerned with the *treatment* of type 1 diabetes. Where in doubt, these decisions were checked by the steering group. The steering group had access to a panel of people with type 1 diabetes, but there were no patient representatives on the steering group itself at this early stage, a situation which was acknowledged as being less than ideal. One of the authors of this paper, RS, became involved in the later stages as a patient representative.

### Our evaluation methods

Building on this original project, we used the data collected by the JLA for a mixed methods analysis with an explanatory sequential design [19]. We began by examining measurable differences between patients, carers and healthcare professionals in terms of the likelihood of their suggested research questions being rejected. We then sought to shed further light on these differences by analysing the content of the rejected suggestions using qualitative methods.

#### Quantitative data analysis

Respondents were coded according to whether or not they had submitted a question which was then rejected and how they had classified their role or relationship with diabetes. Some respondents had dual roles (for example, a person with type 1 diabetes who also had a child with the condition) and were coded positive for each of these categories. Respondents who classified themselves as patients and/or carers were additionally coded as having ‘lived experience’ of type 1 diabetes.

Data were analysed using IBM SPSS Statistics 22. For each respondent group, we calculated the proportion of respondents who submitted a question that was later rejected. Cross-tabulations were used to examine differences in terms of the likelihood of submitting a question that was subsequently rejected outright between the following respondent groups: (a) ‘lived experience’ vs. no ‘lived experience’, (b) healthcare professionals vs. non-healthcare professionals and (c) patients vs. carers (within the ‘lived experience’ category). We did not directly compare patients and carers with healthcare professionals because there was substantial overlap between these roles (33 % of healthcare professionals were also patients or carers).

#### Qualitative data analysis

The aim of qualitative analysis was to gain insight into the kinds of patient and carer (‘lived experience’) submissions that were rejected.

All rejected submissions from carers and patients were analysed by two researchers: JC, an expert in patient involvement but with no relevant experience of long-term conditions, and RS, a service-user-researcher with lived experience of type 1 diabetes. Working individually, JC and RS each developed a coding framework based on emergent themes from the full range of rejected submissions [20]. These interpretations were then compared, discussed and refined until agreement was reached. In a subsequent coding process, themes were grouped into four overarching categories: researchable issues, information about research, personal issues and unclear questions. Data within the large ‘researchable issues’ category were further indexed by theme, with submissions frequently indexed under multiple themes. The quotations in this paper have been specifically chosen to illustrate the categories and themes that emerged from this analysis.

### Co-creation of this study

The research question, design of this study and analysis of existing JLA data were entirely co-created by researchers with relevant skills: JC, a specialist in impact assessment of patient involvement and quantitative data analysis, and RS, a service-user-researcher specialising in qualitative analysis, who has over 25 years experience of living with and self-managing type 1 diabetes. The process of bringing these varied perspectives to a collaborative analysis and discussion of the data was invaluable. Both researchers contributed equally to the project; all elements of the study have therefore been inextricably influenced by this collaboration and mutual involvement. We describe this as a wholly positive partnership based on respect and a genuine interest in each others’ perspectives. SC, who facilitated the original Partnership, acted as advisor to this study and contributed to the write-up of this article along with RS and JC. A lay contributor with over 20 years of experience and qualifications in child and adult education reviewed and revised the article, improving its sense and accessibility to lay readers. As a result of her revisions alone, the Gunning Fog Index (a measure of readability) of the “Plain English summary” decreased from 12.95 to 9.73.

## Results

### Likelihood of submitting a rejected question: differences between respondent groups

A total of 1143 suggestions were submitted by 583 respondents. Each respondent submitted between 1 and 20 suggestions (median 1 suggestion).

By far the largest participating group was people with type 1 diabetes (Table 1). Ninety-nine (17.0 %) respondents classified themselves as having more than one role (patient, carer, healthcare professional, organisation and/or other).

**Table 1:** Respondent roles (*N* = 583)

Role	Total (*N*)	*N* with exclusive role
Patient^a^	401 (68.8 %)	342 (58.7 %)
Carer	108 (18.5 %)	80 (13.7 %)
Healthcare professional	96 (16.5 %)	60 (10.3 %)
Organisation	30 (5.1 %)	2 (0.3 %)
Other^b^	33 (5.7 %)	24 (4.1 %)

^a^People who identify themselves as having type 1 diabetes

^b^Including researchers (*N* = 4), people who identify as having a health condition other than type 1 diabetes (*N* = 2), people who work in health education (*N* = 2), people who work in the health service but do not identify themselves as healthcare professionals (*N* = 1) and people who provided no additional information (*N* = 24)

Two hundred and forty-nine (21.8 %) suggestions from 190 (32.6 %) respondents were classified as ‘not a treatment uncertainty’ and were therefore rejected from further stages of the priority-setting process (Table 2).

**Table 2:** Number of rejected questions, by respondent role (*N* = 249)

Role	Number of rejected questions submitted	Number of rejected questions submitted by respondents with exclusive role
Patient^a^	171 (68.7 %)	143 (57.4 %)
Carer	68 (27.3 %)	58 (23.3 %)
Healthcare professional	27 (10.8 %)	10 (4.0 %)
Organisation	7 (2.8 %)	0 (0.0 %)
Other^b^	11 (4.4 %)	4 (1.6 %)

^a^People who identify themselves as having type 1 diabetes

^b^Including a researcher (*N* = 5), person who identifies as having a health condition other than type 1 diabetes (*N* = 1), person who works in health education (*N* = 1) and people who provided no additional information (*N* = 4)

Respondents with lived experience of type 1 diabetes were significantly more likely to have submitted a question that would be rejected, compared to respondents without lived experience of the condition (Table 3). Moreover, respondents who were healthcare professionals were significantly *less* likely to have submitted a question that would be rejected, compared to respondents who were not healthcare professionals (Table 4). Among respondents with lived experience of type 1 diabetes, carers were significantly more likely than patients to have submitted a question that would be rejected (Table 5).

**Table 3:** Association between lived experience and submission of at least one rejected question (*N* = 583)

	Submitted a question that was rejected	*p* value
Lived experience^a^	175/492 (35.6 %)	<0.0005
No lived experience	15/91 (16.5 %)	

^a^Patients and/or carers, 32/492 (6.5 %) of whom were also healthcare professionals

**Table 4:** Association between healthcare professional role and submission of at least one rejected question (*N* = 583)

	Submitted a question that was rejected	*p* value
Healthcare professionals^a^	22/97 (22.7 %)	0.023
Non-healthcare professionals	168/486 (34.6 %)	

^a^Healthcare professionals, 32/97 (33.0 %) of whom were also patients and/or carers

**Table 5:** Association between patient vs. carer role and submission of least one rejected question (*N* = 475)^a^

	Submitted a question that was rejected	*p* value
Patient (not carer)	129/384 (33.6 %)	0.025
Carer (not patient)	49/91 (53.8 %)	

^a^17 (2.9 %) respondents with a dual patient and carer role were excluded from this analysis

### Rejected questions submitted by respondents with lived experience of type 1 diabetes

The themes that emerged from qualitative analysis of the rejected suggestions from patients and carers were grouped broadly into four categories: researchable issues, research progress, personal issues and unclear questions. In the examples given, all submissions are provided verbatim, with no alterations to the original punctuation or spelling.

### Researchable issues

A small number of submitted questions covered researchable treatment uncertainties to which the answer was ‘already known’, that is, research had already been done and answers found. However, because the JLA protocol dictated that only ‘treatment uncertainties’ should be considered, a far greater number of potentially researchable suggestions were rejected on the basis that they were not considered to be about ‘treatments’.

### Cure

A substantial subsection of these researchable issues was concerned in some way with research for a cure. Although it is difficult to give precise numbers due to issues with wording (discussed below), 13.7 % (34 out of 249) of rejected questions from patients and carers specifically mentioned the word ‘cure(s)’ or ‘cured’, while others made suggestions that could be interpreted as associated with cure research, such as, ‘Will type 1 diabetes ever be solved’. Broad cure questions (for example, ‘can we find a cure?’) were submitted only by those with lived experience of the condition and were routinely rejected. However, when patients and carers came together with healthcare professionals for the final prioritisation workshop in the Partnership process, they expressed concern at the absence of cure questions and asked to include a version of these questions. The Partnership thus reinstated it as an ‘overarching’ priority, although its wording was altered to specify a particular area of research: ‘is stem cell therapy an effective treatment⁄cure?’ [18]. ‘Cure’ was the only type of question reprieved in this way.

### Cause and prevention

Questions about the cause of the disease, and also its prevention, were rejected. Alongside broad requests for research into cause and prevention, some suggested specific environmental factors to explore, such as the use of particular drugs, the presence of pollutants and links with other diseases.

What causes, and therefore leading to what could prevent, type 1 diabetes

Is the cause of type 1 diabetes linked to the use of inhaled steroid asthma inhalers?

Is there a relationsip between autoimune diseases such as type 1 diabetes & hypersensitivity e.g. asthma, peanut allergies etc.?

### Understanding disease

A broad subcategory of the researchable issues rejected fell into the category of ‘understanding disease’. These included research that would help day-to-day self-management: understanding unknown factors that affect fluctuations in blood glucose, investigating risk factors for particular complications in addition to ordinary blood glucose control and looking at the reasons behind particular symptoms or responses to high and low blood glucose.

How can one prevent the liver from producing glucose in stressful situations (when adrenaline is produced)?

Identifying genetics factors associated with early/ late progression to complications

Why do different people react differently to extreme hypos eg/ unconsciousness, coma, seizures?

### Practice and policy

A further subset of questions addressed broader issues such as quality of care, inequalities in care and access to treatment rather than treatments themselves.

Why is advice not the same depending on where you live in the Uk

Why are some hospitals more than others keener on insulin pumps?

Why is there a difference in the UK’s approach to diabetes so different to USA’s?

How many people with diabetes are on an insulin regimen that they feel works for them?

This category also included a number of questions that raised issues around the way people with diabetes are treated by others, including policy makers, healthcare professionals and the general public. For example:

One reads over time […] quite a lot of discrimination against sufferers. It would perhaps seem by “research” methods that this discrimination is uncalled for and could actively be demonstrated to be unfair.

Why do diabetics have so many restrictions put on them in sociaty

Exploration of the policy and economics behind healthcare research and investigation into vested interests that might be slowing research progress were also recurring themes in the rejected questions:

Does type 1 diabetes still exist because there is an investment in keeping the disease going from drug manufacturers?

Why couldnt the Insulin pumps be redeisgned so they are MUCH MUCH LESS EXPENSIVE?

Why it seems to be that advances in this field in treatment, devices as well as in understanding where the disease come from are incredibly slow compared with engineering advances

### Information about research

Not every rejected suggestion contained a question that could be researched. Some were direct requests for information about the process of research or about progress of existing research:

Can more information about the clinical stages of research & trials be explained

What UK in particulary is doing as research for people living with this condition?

Some of these submissions could be considered as prioritising certain areas for research, although they were formulated as questions about existing progress. Thus, the example below might be counted as requesting research into a cure or literally asking for an estimate of the amount of time before a cure can be found:

How soon, realistically, will there be a cure for Type i diabetes?

### Personal issues

A very small number of submissions contained direct questions about issues personal to the respondent’s history or treatment:

What kind of insulin am I on eg Animal?

Again, some of these could have been interpreted more generally, such as the question below, in which the respondent could be asking about specific family circumstances, but could also be raising a more general question about the cause of the disease:

Why did I develop type 1 at 15 (now 65) & my brother at 19 (now 62) with no history in family

### Unclear questions or no question at all

Finally, a few submissions were so broad or difficult to interpret that it was impossible to categorise them with any certainty; some participants openly declared that they did not have a question to submit:

diet and insulin giving more freedom then it does now

I feel quite well informed actually… sorry!

There were also a small number of personal offers to volunteer as a research participant, which were not followed up.

## Discussion

This mixed methods analysis has highlighted a number of important issues to consider when deciding how to involve patients and carers in research priority setting and how to assess the impact of that involvement. Even within the well-respected JLA model examined here, in which involvement takes place in nearly all aspects of the priority-setting process, early decisions about the way questions were framed and what counted as viable suggestions unintentionally reduced patients’ and carers’ potential impact.

In particular, researchers wishing to include lay perspectives should be aware of assuming a shared understanding of the concepts under discussion. In this case study, participants were asked to identify uncertainties about the effects of treatment for diabetes, with the explicit aim of ensuring that the final priority list could be included in a national database available to future researchers and funders [18]. While this had obvious practical advantages, it also had the effect of disempowering respondents with lived experience of type 1 diabetes, and non-healthcare professionals, who were significantly less likely than other respondent groups to contribute research priorities that fit that model.

Analysis of the rejected submissions suggests that ‘treatment’ meant something different to those with lived experience of a chronic condition and that the narrow, medical definition of ‘treatment’ was inadequate to cover the range of other questions which patients and carers wished to raise as priorities for research. Those living with a serious condition regarded it important to initiate research into fair access to treatment (and cure) as well as into the treatment itself. In addition, for patients, treatment may mean ‘the way one is treated’ by others, particularly those with authority or power. It could be argued that the lines between what is a treatment and what is not are much more grey for patients, when a long-term condition is part of every aspect of life, rather than for healthcare professionals or researchers, where it is merely part of one’s working life. To take a hypothetical example, if the effective use of an insulin pump is bound up with how easy it is to get a prescription for that pump, how one’s body reacts to the delivery system in the pump and the way society responds to the person who wants to use the pump, then categorising research priorities by the pump alone conflates many different issues in the lived experience of patients and carers.

A final issue for those engaging with non-researchers in order to develop research questions is to consider what to do with additional requests for information or offers of help. Without a strategy to respond to this kind of submission, valuable opportunities are lost to engage patients in research, answer their questions and make them feel valued.

Our findings are consistent with other priority-setting studies both in the UK and internationally, in which patients have identified a broader range of topics than professionals, including questions related to the environment, psychosocial issues, patient-clinician relationships and service provision [2, 8, 10, 12, 13]. All of these issues, as well as many of those identified in our case study, could be addressed in some way by different disciplines within the broad field of healthcare research, including sociology, psychology, geography and epidemiology. We are also not alone in reporting difficulties interpreting service users’ responses to open-ended questions when there is no opportunity for dialogue or clarification [6]. Such issues may be confounded by service users’ unfamiliarity with research and research programmes’ unfamiliarity with service users [21], risking the unintended distortion or loss of impact. Oliver and colleagues propose that investing time and effort in good communication, training and support should help to overcome this barrier [21]. It could also be argued that service users’ challenge to the concept of what counts as a valid research question is in itself valuable.

In some participatory models of research priority setting, no contributions are rejected without patient influence [12, 13]. The inclusion of service users in rejection decisions could help to close the gap between respondents’ suggestions and the interpretation of these suggestions, as well as increasing their potential impact. In many cases, however, the process by which service users’ suggestions are accepted or rejected is not described or explained and remains a ‘black box’ [22–25]. In the original Priority Setting Partnership, the anonymity of survey submissions meant that further direct dialogue with participants was not possible.

It should be noted that since the completion of the type 1 diabetes project, the JLA itself has made a number of methodological changes to its protocol, including widening of the scope beyond therapeutic research questions, inclusion of the concept of ‘cure’ research and earlier discussion with patients and carers in steering groups about exclusion criteria [16].

A limitation of this study is its focus on a single-question format (‘treatment uncertainties’), single-priority-setting method (the JLA Priority Setting Partnership) and a single condition (type 1 diabetes). We were also unable to adjust for respondent characteristics (e.g. age, ethnicity, education level and socioeconomic status) in our quantitative analysis, as reliable information about these variables was not collected during the original project. It is possible that the observed associations between respondent role and questions being rejected were confounded by such characteristics, and future studies should investigate whether these factors independently influence the impact that patients and carers are able to have.

## Conclusions

This study raises important concerns that are applicable to other service-user involvement initiatives internationally.

Firstly, decisions must always be made at the very start of any research project about its remit, what to include and exclude, and what counts as a legitimate topic for discussion. When these decisions take place before patients and/or carers are involved, researchers may be creating research boundaries which service users might not agree with. They therefore risk limiting or distorting the impact of service-user involvement because the latter’s contribution may not ‘fit’ their pre-determined framework. We recommend that those assessing the impact of service-user involvement examine not only the differences patients and carers make to research but also the impact they *could* have had in the absence of systemic barriers. Without this, it is all too easy to jump to misleading conclusions that service users have no or minimal impact.

Secondly, if such boundaries do have to be created before service users are involved, it is important to define them clearly in accessible language so that service users can maximise the usefulness of their contributions. Similarly, whenever service users’ contributions are rejected, it is important to be transparent about how those decisions are being made and by whom.

Finally, we recommend that service-user involvement initiatives, including research priority-setting partnerships, involve service users as early as possible in collaborative decisions about their design and remit, in order to maximise the potential impact of their contributions. This could lead to service users challenging the very definition of a ‘research question’ and what counts as evidence, and we feel this would be an open debate worth having, mutually beneficial to both service users and researchers in the long run.

## Additional files

Additional file 1: **Survey for collecting suggested research questions.** Copy of the paper survey used by the JLA Priority Setting Partnership to collect treatment uncertainties about type 1 diabetes.

Additional file 2: **Background information sheet for potential survey respondents.** Background information about the type 1 diabetes JLA Priority Setting Partnership for potential survey respondents.

Additional file 3: **Promotional flyer.** Flyer used to promote the survey to potential respondents.

## Abbreviations

JLA: James Lind Alliance
UK: United Kingdom
