# Supplementary material for: Missed opportunities for impact in patient and carer involvement: a mixed methods case study of research priority setting
Source: Res Involv Engagem. 2015 Aug 4;1:7. doi: 10.1186/s40900-015-0007-6 (PMC5611607; doi:10.1186/s40900-015-0007-6)
Supplement: Supplementary file 1 — Survey for collecting suggested research questions. Copy of the paper survey used by the JLA Priority Setting Partnership to collect treatment uncertainties about type 1 diabetes. [file 40900_2015_7_MOESM1_ESM.pdf]

## Type 1 Diabetes Priority Setting Partnership Survey

*Please read the information sheet before you do the survey: go to www.ukdrn.org/JLAsurvey.html or call 01604 622837 to request a copy.*

The Type 1 Diabetes Priority Setting Partnership has been set up by the James Lind Alliance (JLA), the Diabetes Research Network, Diabetes UK, the Insulin Dependent Diabetes Trust, the Juvenile Diabetes Research Foundation, NHS Evidence - diabetes and the Scottish Diabetes Research Network. The Partnership aims to work with people with type 1 diabetes, their carers and clinicians to identify and decide the most important research questions for the treatment of type 1 diabetes.

**Do you have an unanswered question or an uncertainty about the treatment of type 1 diabetes? Do you think that answering that question through research will help to improve the lives of people with type 1 diabetes? This is your chance to make your views known.**

To help you, here are some examples of unanswered questions about other health problems:

- Are breathing exercises helpful in controlling asthma?
- What is the evidence for gargling asprin to relieve a sore throat?
- How safe is it for my baby if I am breastfeeding and taking antidepressant medication?
- Is monochloroacetic acid more effective for the treatment of warts than liquid nitrogen?

**What question(s) about the treatments for type 1 diabetes would you like to see answered by research?** (You can submit as many or as few as you like.)

**1.**

| **2.** |
| --- |
| |

| **3.** |
| --- |
| |

| **4.** |
| --- |
| |

*If you wish to submit more than four unanswered questions, please continue on a separate page.*

**Consent**
As part of this research process, we may publish the question(s) you have identified in the UK Database of Uncertainties about the Effects of Treatments (UK DUETs). Your name or the name of your organisation will **NOT** be published in association with the question(s). Do we have your permission to publish the question(s)? (Please tick one box)

Yes ☐

No ☐

*******************************************************************

**Some questions about you**

It would be really helpful for our research to know a little more about you, so we would be grateful if you could answer the questions in this section. However, if you would prefer not to, then just leave it blank.

**Which of these best describes you? (Please tick all that apply)**

☐ I am a person with type 1 diabetes

☐ I am a carer or relative of someone with type 1 diabetes

☐ I am the parent of a child aged under 16 who has type 1 diabetes

☐ I am a health professional based in primary care

☐ I am a health professional based in secondary care

☐ I am part of an organisation representing people with type 1 diabetes

☐ other – please describe

**<u>You only need to answer this question if you are answering as a clinician or healthcare professional.</u> (Please tick ONE box)**

**In which area do you primarily work?**

☐ Paediatric setting

☐ Adult setting

☐ Both paediatric and adult settings

☐ Other

☐ Not applicable

**<u>You only need to answer these questions if you a person with type 1 diabetes, or if you are the parent, relative or carer of a person with type 1 diabetes.</u>**

These details will NOT be published in association with your response or linked in any way to the UK DUETs database.

**What is your (or the person with type 1 diabetes') age?**

- ☐ 0-9
- ☐ 10-19
- ☐ 20-29
- ☐ 30-39
- ☐ 40-49
- ☐ 50-59
- ☐ 60 or over
- ☐ Prefer not to say

**At what age were you (or the person with type 1 diabetes) diagnosed?**

- ☐ 0-9
- ☐ 10-19
- ☐ 20-29
- ☐ 30-39
- ☐ 40-49
- ☐ 50-59
- ☐ 60 or over
- ☐ Prefer not to say

**Gender: are you (or the person with type 1 diabetes)…**

- ☐ Female
- ☐ Male
- ☐ Prefer not to say

**Ethnic group: are you (or the person with type 1 diabetes)…**

- ☐ White
- ☐ Black
- ☐ Asian
- ☐ Mixed race
- ☐ Chinese or other ethnic group
- ☐ Prefer not to say

**Next steps**

Would you like more information about the next stage of the project, where the research questions will be prioritised?

Yes ☐

No ☐

If you answered 'yes', please supply your contact details.

**Contact details**
Your contact details will be kept confidential and securely, in accordance with the Data Protection Act.

Name ______________________________________________________

Address ____________________________________________________

___________________________________________________________

Email address ______________________________________________

What is your preferred method of contact?

Email ☐

Post ☐

**Thank you for completing this survey.**

Please return this form to:

Duets@nice.org.uk

or

Diabetes Survey
James Lind Alliance
Summertown Pavilion
Middle Way
Oxford OX2 7LG

********************************************************************
