# Supplementary material for: Missed opportunities for impact in patient and carer involvement: a mixed methods case study of research priority setting
Source: Res Involv Engagem. 2015 Aug 4;1:7. doi: 10.1186/s40900-015-0007-6 (PMC5611607; doi:10.1186/s40900-015-0007-6)
Supplement: Supplementary file 2 — Background information sheet for potential survey respondents. Background information about the type 1 diabetes JLA Priority Setting Partnership for potential survey respondents. [file 40900_2015_7_MOESM2_ESM.pdf]

Type 1 Diabetes Priority Setting Partnership

BACKGROUND INFORMATION – please read

The Type 1 Diabetes Priority Setting Partnership has been set up by the James Lind Alliance (JLA), the Diabetes Research Network, Diabetes UK, the Insulin Dependent Diabetes Trust, the Juvenile Diabetes Research Foundation, NHS Evidence - diabetes and the Scottish Diabetes Research Network. The Partnership aims to work with people with type 1 diabetes, their carers and clinicians to identify and decide the most important research questions for the treatment of type 1 diabetes.

Do you have an unanswered question, or an uncertainty about the treatment of type 1 diabetes? Do you think that answering that question through research will help to improve the lives of people with type 1 diabetes? This is your chance to make your views known.

Why is this project important?
Type 1 diabetes occurs when the amount of glucose in the blood is too high. It is a leading cause of kidney failure, adult blindness, stroke, heart attacks and nerve damage leading to amputation. There are many unanswered questions about the treatment of type 1 diabetes. The *NHS Evidence – diabetes 2009 Annual Evidence Update* identified 195 systematic reviews on all aspects of diabetes care, but only 13 of these were exclusively about treatments for type 1 diabetes. Type 1 diabetes is increasing, particularly in the under 5s and therefore it is important to get research evidence on the most effective ways of treating it. The aim of this project is to find out the unanswered questions about type 1 diabetes which **patients**, **carers** and **clinicians** most want answered.

What are 'unanswered questions about treatment'?
They are questions about treatment that cannot be answered by up-to-date information based on research evidence. For this exercise we interpret 'treatment' in its broadest sense, including prevention and testing.

What will happen to the questions?
The questions which are submitted will be **checked** to ensure have not already been answered by reliable research. They will then be combined with questions which have been identified through a review of existing research. These will then be entered into the UK Database of Uncertainties of the Effects of Treatments (UK DUETs – www.library.nhs.uk/duets). They will then go through a process of **prioritisation**, in which you can also get involved, to identify the 10 priorities for research which are of the most importance to patients, carers and clinicians. This list will be published and provided to organisations that fund research.

Why patients, carers and clinicians?
Research on the effects of treatments often overlooks the shared interests of patients, carers and clinicians and other healthcare professionals. The pharmaceutical and medical technology industries and academia play essential roles in developing new treatments, but their priorities are not necessarily the same as those of patients, carers and clinicians. This means areas of potentially valuable research may be neglected.

Who is involved?
Organisations involved in this project include the Diabetes Research Network (DRN), Diabetes UK, the Insulin Dependent Diabetes Trust (IDDT), the Juvenile Diabetes Research Foundation (JDRF), NHS Evidence - diabetes and the Scottish Diabetes Research Network. The JLA is a not-for-profit initiative which brings patients and clinicians together to identify and prioritise uncertainties about the treatments for health problems. The JLA is funded by the National Institute for Health Research and the Medical Research Council.

How can I get involved?
You can participate by completing our survey. It will take you around 5-10 minutes. You can do this in a number of different ways:

- **online**: www.ukdrn.org/JLAsurvey.html
- **email** / **post**: download the survey from www.ukdrn.org/JLAsurvey.html and send to
  - Duets@nice.org.uk
  - JLA, Summertown Pavilion, Middle Way, Oxford OX2 7LG
- **telephone**: call us on 01865 517622 to respond over the phone

You will be asked to give details of all your unanswered questions about type 1 diabetes which you wish to be entered into the prioritisation process. You can enter as many or as few as you like. The survey will also ask you some optional questions about you and, if applicable, your organisation.

The closing date for the survey is 28 May 2010.

Confidentiality and consent
Your contact details will be kept confidential and securely, in accordance with the Data Protection Act. As part of the process, your questions may be published in UK DUETs, and we will need your permission for this to happen. Your name/organisation will NOT be published in association with the question. For further information, or to see an example of what this will look like, go to www.library.nhs.uk/duets or contact us via the details below. The survey is being managed by a full Member of the Market Research Society, whose Code of Conduct will apply.

Contacts
For further information or if you have any comments or complaints about the process, please contact Sally Crowe at the James Lind Alliance:
Email: patkinson@lindalliance.org Phone: 01865 517635
Post: James Lind Initiative, Summertown Pavilion, Middle Way, Oxford OX2 7LG

Useful websites
Diabetes Research Network - www.ukdrn.org
Diabetes UK – www.diabetes.org.uk
Insulin Dependent Diabetes Trust - www.iddtinternational.org
James Lind Alliance – www.lindalliance.org
Juvenile Diabetes Research Foundation - www.jdrf.org.uk
NHS Evidence – diabetes - http://www.library.nhs.uk/diabetes/
Scottish Diabetes Research Network – www.sdrn.org.uk
UK DUETs - www.library.nhs.uk/duets
