# Supplementary material for: Missed opportunities for impact in patient and carer involvement: a mixed methods case study of research priority setting
Source: Res Involv Engagem. 2015 Aug 4;1:7. doi: 10.1186/s40900-015-0007-6 (PMC5611607; doi:10.1186/s40900-015-0007-6)
Supplement: Supplementary file 3 — Promotional flyer. Flyer used to promote the survey to potential respondents. [file 40900_2015_7_MOESM3_ESM.pdf]

# Do you, or a member of your family have type 1 diabetes?

# Are you a healthcare practitioner with an interest in the condition?

## If yes, you can have your say on research into treatments for type 1 diabetes....

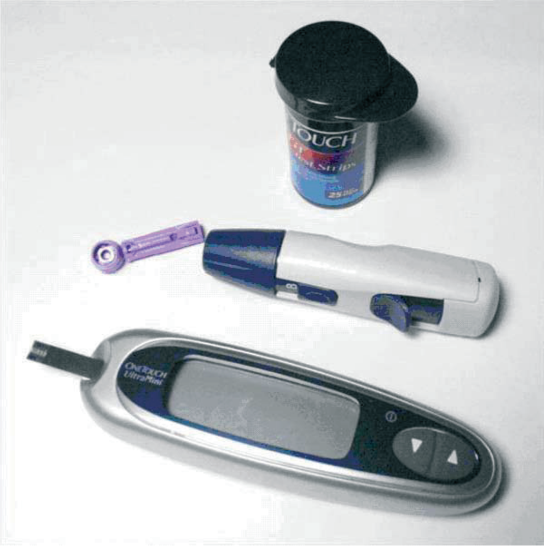

The James Lind Alliance type 1 diabetes Priority Setting Partnership is identifying and deciding the most important research questions for the treatment of type 1 diabetes.

First we need to collect research questions from people living with type 1 diabetes and those caring for them, as well as doctors, nurses, dieticians, and other healthcare practitioners who treat type 1 diabetes.

**Please contribute your treatment research questions visit our survey website**

## www.ukdrn.org/JLAsurvey.html

Paper copy of the survey is available to download. Or contact 01604 622 837 or email enquiries@iddtinternational.org if you would prefer to be sent a paper copy of the survey.

**For other enquiries please email duets@nice.org.uk or call James Lind Alliance on 01865 517635.**

JDRF Juvenile Diabetes Research Foundation *dedicated to finding a cure*

DRN Diabetes Research Network

SDRN Scottish Diabetes Research Network

NHS Evidence

Diabetes UK

The James Lind Alliance
